# Evaluation of effectiveness and safety of the large-format pRESET 6-50 thrombectomy stent-retriever in the endovascular treatment of ischemic stroke: real-world experiences from two tertiary comprehensive stroke centers

**DOI:** 10.3389/fneur.2023.1256365

**Published:** 2023-11-17

**Authors:** Hermann Kraehling, Burak Han Akkurt, Mohamed Elsharkawy, Wolfram Schwindt, Michael Köhler, Nils Werring, Max Masthoff, Anthony Cox, David Minks, Christian Paul Stracke

**Affiliations:** ^1^Department of Radiology, Westfalian Wilhelms-University Münster and University Hospital Münster, Münster, Germany; ^2^Department of Radiology and Department of Interventional Neuroradiology, Westfalian Wilhelms-University Münster and University Hospital Münster, Münster, Germany; ^3^Department of Neurology, Westfalian Wilhelms-University Münster and University Hospital Münster, Münster, Germany; ^4^Department of Interventional Neuroradiology, North Bristol NHS Trust, Bristol, United Kingdom; ^5^Department of Diagnostic and Interventional Neuroradiology, University Medical Center Hamburg-Eppendorf, Hamburg, Germany

**Keywords:** large stent-retriever performance, pRESET 6-50, mechanical thrombectomy, first-pass reperfusion rate, large vessel occlusion (LVO)

## Abstract

**Objective:**

Mechanical thrombectomy (MT) has become the standard treatment for acute ischemic stroke (AIS) with large vessel occlusion (LVO). First-pass (FP) reperfusion of the occluded vessel and fewer passes with stent retrievers show improvement in functional outcomes in stroke patients, while higher numbers of passes are associated with higher complication rates and worse outcomes. Studies indicate that a larger size of the stent-retriever is associated with a higher rate of first-pass reperfusion and improved clinical outcomes. In this retrospective study, we investigated the clinical performance of a recently developed and one of the largest stent-retrievers available in the treatment of LVO (pRESET 6-50, phenox GmbH, Bochum).

**Materials and methods:**

All consecutive patients with ischemic stroke due to proximal large vessel occlusion treated with MT using the pRESET 6-50 stent-retriever in two tertiary stroke centers between 09/2021 and 07/2022 were included in this study. The reperfusion rate after MT was quantified by the modified thrombolysis in cerebral infarction (mTICI) score, and functional neurological outcome was evaluated with the National Institutes of Health Stroke Scale (NIHSS) score and the major early neurological recovery (mENR) rate after 24 h. Successful FP reperfusion was defined as mTICI ≥ 2b. Successful and complete reperfusion were defined as mTICI ≥ 2b and mTICI ≥ 2c, respectively.

**Results:**

In total, 98 patients (52 men and 46 women) with a median age of 75 (range 25–95 years) were included. A total of 70 (72%) patients presented with an occlusion of the middle cerebral artery (MCA) in the M1 segment, 6 (6%) patients with an occlusion of the M2 segment, 17 (17%) patients with an occlusion of the internal carotid artery (ICA), and 5 (5%) patients with an occlusion of the obstructed basilar artery (BA). Successful FP reperfusion was achieved in 58 patients (62%). Successful and complete reperfusion were achieved in 95 (97%) and 82 (83%) patients, respectively. The median National Institutes of Health Stroke Scale (NIHSS) in all treated patients improved from 17 to 7.5. Major early neurological recovery (mENR) was observed in 34 patients (35.1%).

**Conclusion:**

MT with the pRESET 6-50 stent-retriever achieves high successful first-pass and final reperfusion rates in patients with AIS and LVO. The results of this study support the thesis to use large-format stent-retriever in proximal vessel occlusion MT whenever feasible in order to improve high FP and final reperfusion rate, which are known predictors of good clinical outcome.

## Highlights

- MT with the pRESET 6-50 achieves high successful FP reperfusion rates and a high overall rate of reperfusion in patients with AIS and LVO.

## Introduction

Mechanical thrombectomy (MT) has become the gold standard in the treatment of acute ischemic stroke (AIS) due to large vessel occlusion (LVO) (1, 2). MT is based on the retrieval of the occluding clot with stent-retrievers enabling fast recanalization of the affected vessel and full restoration of blood flow. These stent-retriever-based techniques were assessed in large controlled randomized trials, which demonstrated superior efficacy of MT in LVO regarding patient outcomes compared to intravenous thrombolysis (IVT) alone (3–6).

Complete reperfusion, measured by the modified Thrombolysis in Cerebral Infarction Score (mTICI), constitutes the main goal of MT. While successful recanalization (mTICI ≥ 2b) was an initial endpoint in several trials, higher reperfusion rates lead to superior clinical outcomes, and full reperfusion (mTICI 3) is associated with improved neurological status and better functional outcomes (7, 8).

In addition, first-pass (FP) reperfusion forms an indicator of a good clinical outcome and is therefore assessed in many clinical trials investigating the performance of thrombectomy devices (9, 10).

Meanwhile, a great variety of stent-retrievers with different shapes, sizes, and materials from various vendors have been introduced for MT. While the selection of the appropriate device currently remains at the discretion of the performing neurointerventionalist depending on the individual circumstances, larger stent-retriever devices have been associated with a higher complete reperfusion rate and better clinico-neurological outcomes and therefore might be recommended in AIS with LVO (11, 12). The clinical performance of similar stent-retrievers has been investigated in previous studies, showing FP rates of 34.8% (Tigertriever XL) and 40.5% (Solitaire) (13, 14).

This retrospective, multi-center study aimed to evaluate the safety, technical effectiveness, and clinical performance of one of the largest stent-retrievers available, the pRESET 6-50 (phenox GmbH, Bochum, Germany), applied to patients suffering from AIS with LVO.

## Materials and methods

**Figure 1:**
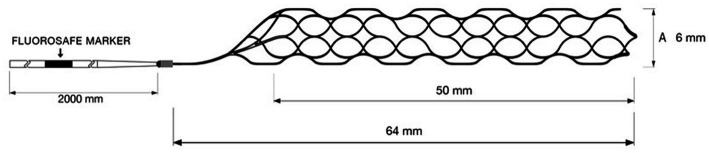
The pRESET 6-50 stent-retriever is one of the largest commercially available stent-retrievers on the market today.

We retrospectively studied a consecutive patient cohort of 98 patients who met the inclusion criteria and underwent mechanical thrombectomy with the pRESET 6-50 stent-retriever (Figure 1) at two tertiary stroke centers between September 2021 and July 2022.

This study included 98 patients (52 men and 46 women) with a mean age of 71.4 (± 15.5) years who received a mechanical thrombectomy with a pRESET 6 × 50 stent-retriever within the study period. A total of 70 (71%) patients presented with vascular occlusion of the M1 segment of the middle cerebral artery (MCA-M1), 6 (6%) patients with an occlusion of the M2 segment of the middle cerebral artery (MCA-M2), 17 (17%) patients presented with an occlusion of the internal carotid artery (ICA), and 5 (5%) patients presented with an occlusion of the basilar artery (BA). Systemic lysis therapy was performed in 47 (48%) patients. The median NIHSS at admission was 17 (range 3–38).

The demographic and baseline characteristics of the study cohort can be found in Table 1.

**Table 1:** Demographic and baseline characteristics.

	Overall	Center 1	Center 2
Number of patients	98	67	31
Age (in years, mean ± SD)	71.4 ± 15.5	71.5 ± 15.8	71.2 ± 15.1
Sex (male/female)	52/46	36 / 31	16/15
NIHSS at admission (median, range)	17 (3–38)	17 (3–38)	19 (7–28)
**Occluded vessel**			
M1-MCA, total no (%)	70 (71)	49 (73)	21 (68)
M2-MCA, total no (%)	6 (6)	5 (7)	1 (3)
ICA, total no (%)	17 (17)	9 (13)	8 (26)
BA, total no (%)	5 (5)	4 (6)	1 (3)
Preinterventional iv. lysis total no (%)	47 (48)	33 (49)	14 (45)

M1-MCA, middle cerebral artery M1 segment; M2-MCA, middle cerebral artery M2 segment; ICA, internal carotid artery; BA, basilar artery; NIHSS, National Institutes of Health Stroke Scale; total no, total number; min, minutes; SD, standard deviation.

### Study population

The inclusion criteria were the presence of cerebral vessel occlusion (I), the performance of thrombectomy using the pRESET 6-50 stent-retriever (II), an assessment of the success of thrombectomy using the modified thrombolysis in cerebral infarction score mTICI (III), and the presence of a post-interventional neurological assessment based on the use of major early neurological recovery (mENR) and the National Institutes of Health Stroke Scale (NIHSS) (IV) (15, 16).

### Diagnostic workflow

The preinterventional diagnosis of AIS with LVO was made in the investigating centers by computed tomography (CT), CT angiography (CTA), and CT perfusion (CTP). The presence or absence of a mismatch in CTP was helpful for further decision-making on thrombectomy but was not a definitive exclusion criterion. Patients transferred from external hospitals had been examined there by CT or magnetic resonance imaging (MRI). If there were no contraindications to intravenous lysis therapy based on the initial imaging diagnosis and clinical history, it was initiated immediately according to national guidelines with both centers using the same regimen [0.9 mg/kg alteplase per kilogram body weight, max 90 mg/60 min (1, 17)].

The National Institutes of Health Stroke Scale (NIHSS) was used to assess the extent of neurological impairment pre-interventionally, 24 h after thrombectomy, and at the time of discharge by colleagues of the neurological department (18).

The time (min) that elapsed from the onset of neurological symptoms to the first CT or MR imaging was evaluated (onset-to-imaging), as was the time between imaging and the start of thrombectomy (onset-to-groin-puncture) and the total duration of the thrombectomy procedure (groin-puncture-to-reperfusion; Table 2).

**Table 2:** Site-specific results of thrombectomies with the pRESET 6-50 stent-retriever.

MT technical outcome and evaluation	Overall	Center 1	Center 2
Stroke-to-image min (mean ± SD)	180 ± 216	187 ± 233	162 ± 163
Stroke-to-puncture, min (mean ± SD)	344 ± 250	301 ± 244	461 ± 233
Puncture-to-reperfusion, min (mean ± SD)	46 ± 28	46 ± 30	45 ± 24
First-pass success (mTICI ≥ 2b) *n*, %	61 (62)	44 (66)	17 (55)
Succesful recanalization (mTICI ≥ 2b) *n*, %	95 (97)	64 (95)	31 (100)
Complete recanalization (mTICI ≥ 2c) *n*, %	82 (83)	61 (91)	21 (68)
NIHSS (median) at Admission	17	17	19
NIHSS (median) 24 h post treatment	11	10.5	12
NIHSS (median) at discharge	7.5	6	10
Post treatment parenchymal hemorrhage *n*, %	21 (21)	13 (19)	8 (26)
Post treatment sICH (ECASS III) *n*, %	5 (5)	3 (3)	2 (2)

MT, Mechanical Thrombectomy; mTICI, Modified treatment in cerebral ischemia; NIHSS, National Institutes of Health Stroke Scale; sICH, symptomatic intracerebral hemorrhage; ECASS III, European Cooperative Acute Stroke Study. Technical characteristics (technical thrombectomy attempt) did not have a significant influence on first-pass success, successful recanalization, complete recanalization, post treatment parenchymal hemorrhage and post treatment sICH (all values *p* > 0.05).

### Mechanical thrombectomy technique and technical outcome evaluation

MT was performed in both centers by trained and certified interventional neuroradiologists with at least 8 years (M.E. 8 years, W.S. 15 years, M.K. 15 years, D.M. 8 years, and C.P.S. 17 years) of professional experience in the investigating centers.

**Figure 2:**
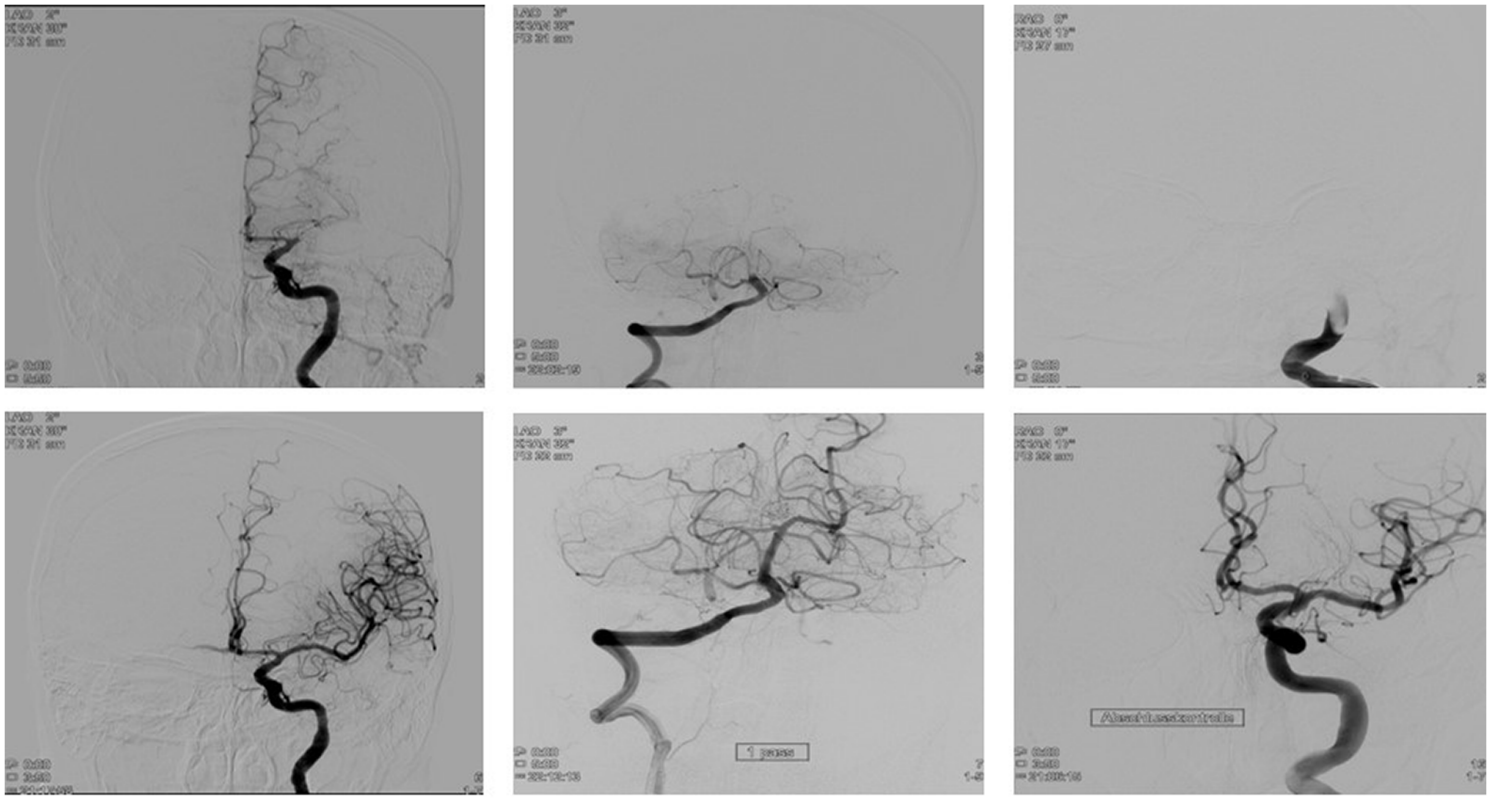
Mechanical thrombectomy (MT) with the pRESET 6-50 stent-retriever in patients with large vessel occlusion (LVO). Mt with the pRESET 6-50 achieves high successful first pass reperfusion in patients LKVO in the middle cerebral artery **(A)**, the basilar artery **(B)**, and the internal carotid artery **(C)**.

In all cases, MT was performed via an arterial transfemoral approach under general anesthesia (Figure 2). Devices and catheter material besides the pRESET 6-50 stent-retriever for MT differed among the two centers.

Center 1 used the following materials as standard for MT: 8F sheath, flowgate balloon catheter + vertebralis select cathether (Stryker™, Fremont, United States), Terumo standard wire (Radiofocus™, Tokyo, Japan), Rebar 18 microcatheter (Medtronic™, Irvine, United States, 0.021-inch inner diameter), HeadwayDuo microcatheter (Microvention™, Tustin, United States, 0.0165-inch inner diameter), Traxcess 14 microwire (Microvention™, Tustin, United States), and VacLok AT Vacuum Syringe (Merit Medical™, South Jordan, United States).

Center 2 used the following materials as standard for MT: 8F sheath, NeuronMax (Penumbra™, Alameda, United States) or Infinity Plus (Stryker™, Fremont, United States), Sim Select (Penumbra™, Alameda, United States), Terumo Advantage (Radiofocus™, Tokyo, Japan), Vecta 74 (Stryker™, Fremont, United States), Velocity (Penumbra™, Alameda, United States), Synchro-14 or Synchro Select (Stryker™, Fremont, United States), VacLok AT Vacuum Syringe (Merit Medical™, South Jordan, United States), Stryker Medelo vacuum pump (Stryker™, Fremont, United States), or Penumbra Engine vacuum pump (Penumbra™, Alameda, United States).

The afterhand use of other stent-retrievers was not an exclusion criterion in both centers.

The reported first-pass effect refers to pRESET 6-50 alone. Final reperfusion scores are attributed to all maneuvers during the entire procedure. Therefore, they include a change to other retrievers after failed or incomplete reperfusion with pRESET 6 × 50 or afterhand use of smaller devices in residual peripheral occlusions.

While in Center 1, MTs were performed with a coaxial thrombectomy technique (balloon catheter + stent-retriever), MTs in Center 2 were performed with a triaxial thrombectomy technique (guide catheter + intermediate catheter + stent-retriever).

A first-pass (FP) success thrombectomy was defined as reperfusion (≥mTICI 2b) of the occluded vessel with a single pass of the pRESET 6-50 stent-retriever. Successful recanalization was defined as a final reperfusion score of ≥ mTICI 2b, whereas complete recanalization was defined as a final reperfusion score of ≥ mTICI 2c. The numbers of passes performed with pRESET 6-50 stent-retriever during MTs and the final mTICI score were recorded.

### Postinterventional neurological evaluation

Postinterventional neurological status was evaluated by neurologists with at least 5 years (N.W. Center 1, stroke physicians and advanced nurse practitioners, Center 2) of professional experience in the investigating centers. The functional neurological outcome was evaluated by the rate of early major neurological recovery (mENR), defined as a decrease of the baseline NIHSS of at least eight points or reaching an NIHSS of 0–1 according to HERMES classification (19).

The study was approved by the local ethics committee (AZ: 2022-243-f-S). Informed consent from patients was waived due to the retrospective nature of this study.

### Statistical analysis

Data were presented as means (± standard deviation), medians (with range, minimum, and maximum), or absolutes and percentages, depending on the nature of the available data. The Shapiro–Wilk test was used to analyze the data for a normal distribution. A groupwise comparison of both patient cohorts at the respective centers was performed. Univariate logistic regression was performed to identify potential independent baseline parameters associated with thrombectomy outcome scores (FP, Menr). The Friedman test was performed for the analysis of neurological outcomes measured by NIHSS scores at admission, 24 h after thrombectomy, and at discharge.

Statistical testing was performed using SPSS (version 28.0.1.1, IBM Corp., United States), with a value of *p* of <0.05 considered statistically significant.

## Results

Overall, our cohort is similar to the interventional population in the HERMES data cohort in terms of baseline data (20). We included *n* = 98 patients Center 1: *n* = 67, Center 2: *n* = 31, 52 men (53%, HERMES 52%), 46 women (47%, HERMES 48%), with a median age of 75 years (range 25–95, HERMES 68, range 57–77).

A total of 70 patients (71%, HERMES 69%) presented with vascular occlusions of the M1 segment of the middle cerebral artery (MCA-M1), and 6 patients (6%, HERMES 8%) presented with occlusions of the M2 segment of the middle cerebral artery (MCA-M2). In total, 17 patients (17%, HERMES 21%) presented with an occlusion of the internal carotid artery (ICA), and 5 patients (5%, HERMES 2%) presented with an occlusion of the basilar artery (BA).

The baseline median NIHSS at admission was 17 (range 3–38; HERMES 17, range 14–20). Preinterventional lysis therapy was applied to a comparable extent in both hospitals (Center 1: 49%, Center 2: 45%, HERMES 83%). The collected data were analyzed for each site and summarized/evaluated cumulatively, as shown in Table 2.

### MT: technical outcome evaluation

The time elapsed from the onset of neurologic symptoms to the first CT or MR imaging (onset-to-imaging) was 103 min (min) median (mean 180 ± 216 min), Center 1: median 95 min (mean 187 ± 233 min), and Center 2: median 109 min (mean 162 ± 163 min).

The time from initial imaging to the start of thrombectomy (onset-to-groin-puncture) was a median of 135 min (mean 189 ± 208 min), Center 1: median 68 min (mean 118 ± 130 min), and Center 2: median 275 min (mean 341 ± 259 min).

The median duration of the thrombectomy procedure (groin-puncture-to-reperfusion) was 38 min (mean 46 ± 28 min), Center 1: median 36 min (mean 46 ± 30 min), and Center 2: median 42 min (mean 45 ± 24 min).

The average number of passes to achieve successful reperfusion was 1.5 (range 1–6).

Regarding the technical outcomes of the pRESET 6-50 stent retriever, the first-pass success rate (recanalization ≥ mTICI 2b) was 62% (61 patients; Center 1: 66%, Center 2: 55%). Successful recanalization (≥mTICI 2b) of the occlusion was achieved in 95 patients (97%; Center 1: 95%, Center 2: 100%). Complete recanalization (≥mTICI 2c) of the occlusion was achieved in 82 patients (83%; Center 1: 91%, Center 2: 68%). Supportive therapy following successful thrombectomy (e.g., treatment of a stenosis of the internal carotid artery with a stent) was necessary in 16% of cases (Center 1: 18%, Center 2: 13%).

### MT: safety evaluation

In total, there was only one case of material failure (1%) during the interventional use of the pRESET 6-50 as the stent-retriever could not be deployed. The material failure did not have any negative consequences for the patient (Center 1). In all other 97 (99%) deployments, the pRESET 6-50 stent-retriever was used without the occurrence of any technical or material-related problems. No intraprocedural hemorrhage occurred during MTs.

### Clinical outcome evaluation

The median baseline NIHSS at admission was 17 points (range 3–38), which improved to 11 points 24 h after thrombectomy. The median NIHSS at discharge was 7.5. The overall median improvement in NIHSS was 9.5 points (Center 1: 11 points, Center 2: 9).

The rate of mENR after thrombectomy with the pRESET 6-50 stent-retriever was 35%.

The post-interventional occurrence of new ischemia in another part of the brain not attributable to the initially occluded cerebral vascular territory was detected via CT in a total of only four cases (4%, Center 1: two cases, Center 2: two cases).

Compared to the initial imaging, 21 patients presented with a new hemorrhage in postinterventional CT, while in 5 patients (5%), symptomatic intracerebral hemorrhage (sICH) occurred with a worsening of the NIHSS of more than four points (Center 1: 3 patients, Center 2: 2 patients) (21).

Univariate logistic regression showed no statistical influence of baseline characteristics on FPE or mENR [age (*p* = 0.427/0.078), gender (*p* = 0.055/0.306), preinterventional lysis (*p* = 0.070/0.507), NIHSS at admission (*p* = 0.056/0.852), and occluded vessel (*p* = 0.094/0.190)].

Baseline characteristics were similar in both cohorts. Onset-to-groin-puncture (301 ± 244 min vs. 461 ± 233 min, *p* = 0.008) and complete recanalization with mTICI ≥ 2c (91% vs. 68%, *p* = 0.010) were significantly different between both groups.

The analysis of the development of the NIHSS score from admission to 24 h post-intervention to discharge showed significant differences. Compared to NIHSS at admission (median 17, 3–38), there was a significant improvement of the score immediately after thrombectomy at 24 h (11, 0–38; *p* < 0.001) and at discharge (7.5, 0–38, *p* < 0.001).

## Discussion

This study aimed to investigate the effectiveness and safety of the pRESET 6-50 stent-retriever for MT in AIS with LVO.

The pRESET 6-50 stent-retriever showed a high first-pass success rate (62%), a high success rate in terms of the overall success of thrombectomy (97%), and subsequently achieved significant clinical improvement for the treated patients (mENR).

Our study supports evidence from previous studies that the use of a larger stent-retriever in thrombectomy leads to an improved first-pass rate and better post-interventional clinical outcomes (22). The assumption that a large stent-retriever offers a larger surface area for the integration of the retriever into the clot due to its greater working length and thus leads to higher first-pass effects has already been discussed by other studies and is in line with our experience (11, 12, 23). Studies investigating the performance of other available large-format stent retrievers show similar results, indicating safe and effective clinical use. Maus et al. reported a successful reperfusion rate (mTICI 2b-3) of 78.3% in LVO by using the Tigertriever XL. Further studies comparing the performance of the 4 mm- and 6 mm-diameter Solitaire stent retrievers did not find differences in outcomes of endovascular treatment (12, 24). *In vitro* studies examining the clot removal of longer stent retrievers have previously shown high rates of complete recanalization (25, 26).

Thus, in LVO, the largest possible stent-retriever should be chosen for the first thrombectomy attempt (25, 27).

Previous studies showed that a high number of needed passes for successful recanalization is associated with higher rates of complications such as bleeding and worse clinical outcomes (28, 29). The pRESET 6-50 stent-retriever, in addition to its high FP success rate, also demonstrates a very high rate of successful recanalization (mTICI ≥ 2b). In fact, only three thrombectomy attempts are needed to achieve successful recanalization in 97% of cases (62% with the first thrombectomy attempt, an additional 25% after the second thrombectomy attempt, and an additional 6% after the third thrombectomy attempt). The average number of thrombectomy attempts required for successful recanalization was 1.5. Our results further show slightly better FP success rates and clinical outcomes in Center 1 compared with Center 2. This may be due to the coaxial thrombectomy technique mainly used in Center 1 and the associated shorter time required for successful thrombectomy.

A specific major concern against the use of very large stent-retrievers is the possible higher risk of severe bleeding. Our study demonstrates that despite its large size, pRESET 6-50 does not have an increased risk of periinterventional bleeding. This is consistent with the results of previous *in vitro* studies for 6-mm diameter stent-retrievers (23). In fact, the overall risk of bleeding when using the pRESET 6-50 stent-retriever is 21%, whereas only 5% sICH occurred, which is within the range reported for other, smaller stent-retrievers such as in the HERMES meta-analysis (4.4%) (20, 30).

The authors are well aware of the limitations of this study: This retrospective study consists of a consecutive cohort of patients in two different centers in two countries without exact records of comorbidities. During the inclusion period, different retrievers were available in both centers, and the use of the pRESET 6-50 was up to the discretion of the neurointerventionalist. Certain inhomogeneities within the patient collective must be taken into account with regard to the post-interventional development in relation to the underlying, original health condition of the patient.

An exact comparison between the performance of thrombectomies is *per se* difficult when several operators with possibly somewhat different experience and technical skills are involved. Despite the different techniques and intervention materials used in the thrombectomies, the results regarding the first-pass success and the post-interventional clinical development of the patients show that the pRESET 6-50 stent-retriever can lead to good results relatively independent of the respective technical approach.

As shown by previous studies, success rates of the first-pass effect using a coaxial thrombectomy technique are comparable to those using a triaxial thrombectomy technique (31). Since exclusively only one form of thrombectomy technique was used in the investigated centers, it can be assumed that there is a high level of experience regarding the respective technique at both sites.

The results in terms of achieved mTICI scores, successful first-pass rates, and post-interventional neurological outcomes demonstrate the high efficacy of the pRESET 6-50 stent-retriever in the treatment of LVO, regardless of the location of the occluded vessel.

## Conclusion

Our study shows that the pRESET 6-0 stent-retriever can be used successfully and safely for the treatment of AIS due to proximal cerebral vessel occlusion, with high first-pass success rates and good clinical-neurological outcomes. However, the findings should be interpreted in the context of its retrospective design and the mentioned procedural variations. Further prospective evaluation of the device and its efficacy relative to standard stent retrievers is recommended.

## Data availability statement

Clinical data is available on reasonable request. Requests to access these datasets should be directed to hermann.kraehling@ukmuenster.de.

## Ethics statement

The studies involving humans were approved by Ethics Committee of the University of Münster. The studies were conducted in accordance with the local legislation and institutional requirements. The ethics committee/institutional review board waived the requirement of written informed consent for participation from the participants or the participants’ legal guardians/next of kin because of the retrospective nature of the studytement.

## Author contributions

HK: Conceptualization, Data curation, Formal Analysis, Investigation, Methodology, Project administration, Visualization, Writing – original draft, Writing – review & editing. BA: Conceptualization, Data curation, Formal Analysis, Investigation, Methodology, Project administration, Visualization, Writing – original draft, Writing – review & editing. ME: Investigation, Supervision, Writing – review & editing. WS: Investigation, Supervision, Writing – review & editing. MK: Investigation, Supervision, Writing – review & editing. NW: Data curation, Formal Analysis, Writing – review & editing. MM: Formal Analysis, Investigation, Writing – review & editing. AC: Data curation, Formal Analysis, Investigation, Writing – review & editing. DM: Data curation, Formal Analysis, Investigation, Project administration, Supervision, Validation, Writing – review & editing. CS: Conceptualization, Data curation, Formal Analysis, Investigation, Methodology, Project administration, Resources, Supervision, Validation, Writing – review & editing.
